# Evolutionary reversion in tumorigenesis

**DOI:** 10.3389/fonc.2023.1282417

**Published:** 2023-11-07

**Authors:** Yosuke Nagahata, Hiroshi Kawamoto

**Affiliations:** Laboratory of Immunology, Institute for Life and Medical Sciences, Kyoto University, Kyoto, Japan

**Keywords:** tumorigenesis, evolution, unicellular organism, multicellularization, cross-species comparison, transcriptome

## Abstract

Cells forming malignant tumors are distinguished from those forming normal tissues based on several features: accelerated/dysregulated cell division, disruption of physiologic apoptosis, maturation/differentiation arrest, loss of polarity, and invasive potential. Among them, accelerated cell division and differentiation arrest make tumor cells similar to stem/progenitor cells, and this is why tumorigenesis is often regarded as developmental reversion. Here, in addition to developmental reversion, we propose another insight into tumorigenesis from a phylogeny viewpoint. Based on the finding that tumor cells also share some features with unicellular organisms, we propose that tumorigenesis can be regarded as “evolutionary reversion”. Recent advances in sequencing technologies and the ability to identify gene homologous have made it possible to perform comprehensive cross-species transcriptome comparisons and, in our recent study, we found that leukemic cells resulting from a polycomb dysfunction transcriptionally resemble unicellular organisms. Analyzing tumorigenesis from the viewpoint of phylogeny should reveal new aspects of tumorigenesis in the near future, and contribute to overcoming malignant tumors by developing new therapies.

## Developmental reversion in tumorigenesis

**Figure 1:**
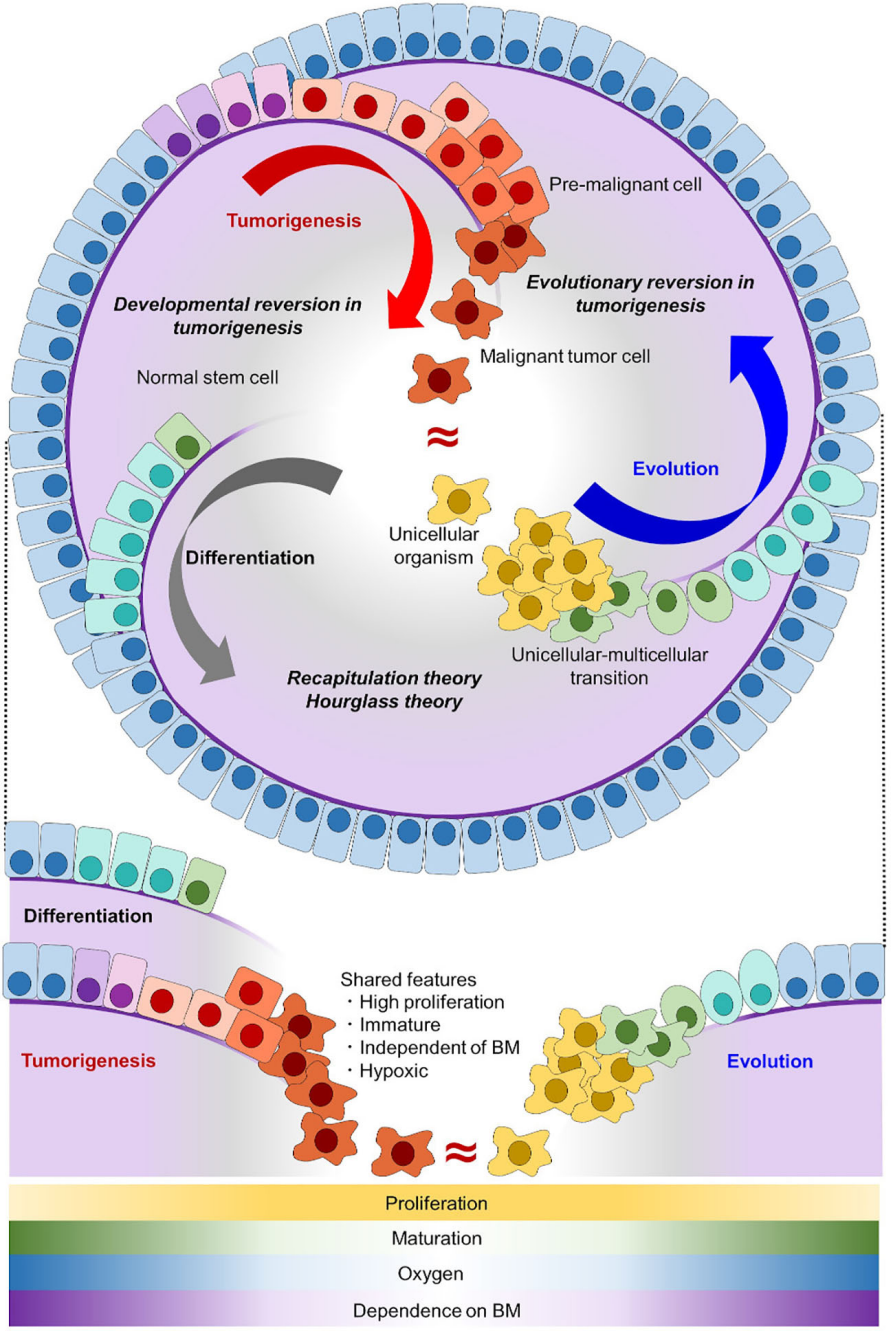
Tumorigenesis can be well explained as an evolutionary reversion. In the normal differentiation of epithelial cells (gray arc arrow), tissue stem cells with self-renewal potential generate differentiated progeny cells. In tumorigenesis (red arc arrow), malignant tumor cells acquire self-renewal potential and have an immature phenotype, which makes tumor cells similar to stem cells. In the evolutionary history of early animals (blue arc arrow), ancient unicellular organisms had no basement membrane (purple line) and they proliferated indefinitely. With the onset of multicellularization, early multicellular animals evolved epithelial cells with a basement membrane. Normal stem cells share the proliferative capacity and immature phenotype with malignant tumor cells, but not the invasive potential through a basement membrane. Unicellular organisms share all of these features with malignant tumor cells. Shared features between differentiation/development and evolution are conceptualized as recapitulation theory and hourglass theory (9, 10), and inversely shared features between differentiation/development and tumorigenesis are conceptualized as developmental reversion in tumorigenesis. Here, we proposed that inversely shared features between evolution and tumorigenesis can be conceptualized as evolutionary reversion in tumorigenesis. BM, basement membrane.

Cells forming malignant tumors are distinguished from those forming normal tissues based on several features. The first is accelerated/dysregulated cell division with disruption of physiological apoptosis, which makes it easy for malignant cells to proliferate and difficult for them to die (1, 2). The second and third are maturation/differentiation arrest and loss of polarity, which make malignant cells different from normal cells in appearance, and enables us to make pathological diagnoses including dysplasia (3, 4). The fourth is invasive potential, which enables malignant cells to invade through the basement membrane and spread to other organs resulting in metastasis (2, 5, 6). Among these features, accelerated cell division and maturation/differentiation arrest make malignant tumor cells similar to normal undifferentiated cells or stem/progenitor cells, and thus tumorigenesis is often regarded as a reversion of differentiation (7, 8) (**Figure 1**).

The grade of undifferentiated status is clinically important because it is a prognostic factor in some malignant tumors such as thyroid tumors (11) and acute myeloid leukemia (AML) (12–14); undifferentiated tumors are typically more malignant and have an unfavorable prognosis. One limitation of studies until a few decades ago was that comparison was based on the microscopic appearance of tissue sections or cell smear samples, or on expression profiles of genes of probable interest by reverse-transcription PCR or microarray experiments. Although these classifications are valuable and have resulted in improving clinical outcomes, they are neither unbiased nor comprehensive evaluations, and the possibility of unintended bias cannot be completely excluded.

Recent advances in the analysis of cell status with next-generation sequencing, e.g., RNA sequencing, make it possible to evaluate cell status comprehensively without or at least with minimum bias. We are now able to comprehensively compare grades of undifferentiated status of different cells. Some malignant tumor-specific gene expression profiles have been revealed by comparing transcriptome data of cancer cells and normal cells using RNA sequencing technologies (15). In AML, transcriptomic analysis with RNA sequencing data confirmed that undifferentiated leukemia has a worse prognosis (16–18). These technological advances firmly established that malignant tumor cells have some aspects of reversion of differentiation even when evaluated comprehensively (19, 20). However, while normal stem cells and tumor cells share features such as accelerated proliferation and maturation/differentiation arrest, these two cell types are definitively different in that the latter cells threaten our lives. In other words, reversion in differentiation does not fully explain tumorigenesis and, therefore, it is still important to seek other aspects of tumorigenesis in order to completely understand what malignant tumor cells are and how they arise.

When malignant tumor cells and normal stem cells or benign tumor cells are compared, the invasive potential is an essential feature of the malignant cells. Indeed, this feature makes malignant tumor cells “malignant”; tumor cells invade through a basement membrane and some of them metastasize into other organs (21, 22). In cases with metastatic lesions, patients cannot be cured even if the primary tumor is surgically resected. Loss of polarity is another important feature and pathologist often make a diagnosis based on this (23, 24). Loss of cell polarity is also found in cells in dysplastic tissue, which can be regarded as a premalignant state. Collectively, tumorigenesis cannot be explained simply by the phenomenon of developmental reversion.

## Malignant tumor cells share some features with unicellular organism

Here, in addition to developmental reversion, we propose another insight into tumorigenesis from the viewpoint of evolution and phylogeny. When malignant tumor cells and normal cells are compared with unicellular organisms, the tumor cells share more features in common. In an adequate environment, typical unicellular organisms divide indefinitely, lack a basement membrane, and have no/less cell polarity compared with epithelial cells of animals (25) (**Figure 1**). Among them, lacking basement membrane or invading through it enables unicellular organisms and malignant tumor cells to be distinguished from normal stem/progenitor cell. As invasive potential is an essential for malignancy, evolutionary reversion explains tumorigenesis better than developmental reversion. Together with the fact that multicellular animals including *Homo sapiens* evolved from an ancestral unicellular organism, it is possible to conceptualize that tumorigenesis represents an evolutionary reversion (**Figure 1**). This evolutionary model in tumorigenesis is different form a traditional tumor micro-evolution model. While the traditional model focuses on tumor evolution in individuals, our model focuses on similarities between tumor cells and unicellular organisms from a view point of phylogeny. Although these two models differ, they can complement each other, and help us understand tumorigenesis more precisely.

Up to now, there are few studies reporting a relationship between tumorigenesis and phylogenetic evolution. Davies and Lineweaver suggested that cancer resembles a prototypic multicellular animal because cancer loses systematic regulation and differentiation (26). In line with this report, Chen et al. reported that the emergence rate of cancer drivers peaked on the deepest branches of multicellular animals, thus, cancer drivers are ancient genes (27). Limitations of this study are that it focused only on tumor-related genes and that only the numbers of genes were examined, while expression levels were not evaluated. Following the study, Trigos et al. and Zhou et al. challenged this limitation. They performed a comprehensive analysis and found that tumor cells more highly expressed these ancient genes compared with normal cells (28–31). Even though a relationship between tumorigenesis and phylogenetics has thus been strongly suggested, another important limitation still remains. These earlier studies compared transcriptional data only among human cells, but not between human cells and unicellular organisms. In other words, it has not been evaluated whether tumor cells truly resemble unicellular organisms in terms of transcriptional profiles, and such a cross-species comparison is needed to make a more precise conclusion.

Another issue has also emerged; some unicellular organisms are not simply unicellular but already have features of multicellularity (32, 33). Whereas it has been suggested in several studies that malignant tumor cells mimic those of early multicellular animals (26, 27, 30), recent advances in the phylogenetics of unicellular and multicellular organisms identified a novel phase for the initiation of multicellularization. Some unicellular organisms display an aggregative state, which should be the prototype of multicellularity (32–42), and cells of some multicellular animals show plasticity like unicellular organisms (43). Thus, it has become possible and important to investigate what it is that malignant tumor cells resemble: early multicellular animals, unicellular organisms, or both.

## Comprehensive cross-species comparison of transcriptome data

What makes it difficult to perform cross-species comparisons of transcriptome data is that different species have different genomes. In order to overcome this issue, homologous genes in different species, homologs or orthologs, in other words, should be identified among all genes and throughout all species. Only then can transcriptome data of different species be comprehensively and objectively compared as in a usual analysis of one species (44–46). Recently, by using the OrthoFinder algorithm (47), we performed a cross-species transcriptomic analysis by focusing on four species: mouse, tunicate, sponge, and *Capsaspora owczarzaki*, a unicellular organism (**Supplementary Figures 1A, B**). Based on such analysis, we succeeded in tracing the evolutionary history of blood cells to the unicellular ancestor of animals (48).

In that study, we have succeeded in comparing transcriptome data from a viewpoint of both phylogenetics and intra-species lineages. In other words, inter-species lineage analysis was performed by comparing data among i) phagocytic cells, ii) non-phagocytic blood cells, and iii) non-blood cells. We found that macrophages in mouse, tunicate, and sponge are transcriptionally similar to each other and to *C. owczarzaki* (**Supplementary Figure 1C**). This similarity indicated that macrophage-like phagocytes were the initial blood cells of animals and that their origin can be traced back to unicellular organisms: a common ancestor of animals and *C. owczarzaki*.

When mechanisms of multicellularity are focused on, cell-cell adhesion is a typical feature in multicellularity, and cadherin plays an important role (49–51). Based on our analysis, *C. owczarzaki* does not have cadherin homologs, but proto-cadherin homologs were identified. This proto-cadherin homologous group also contains FAT family genes, which are known to be tumor suppressors and play roles in maintaining cell polarity (52, 53). Interestingly, within *C. owczarzaki*, expression levels of proto-cadherin homolog are higher in the aggregative stage than in the filopodial (amoeboid) stage (**Supplementary Figure 1D**) (unpublished data obtained by re-analyzing the dataset in our recent report) (48). These results support the idea that the aggregative stage of *C. owczarzaki* represents an intermediate state between unicellular and multicellular organisms, and also support our hypothesis that tumorigenesis has some aspects of loss of multicellularization; i.e. evolutionary reversion.

## Evolutionary reversion in tumorigenesis

**Figure 2:**
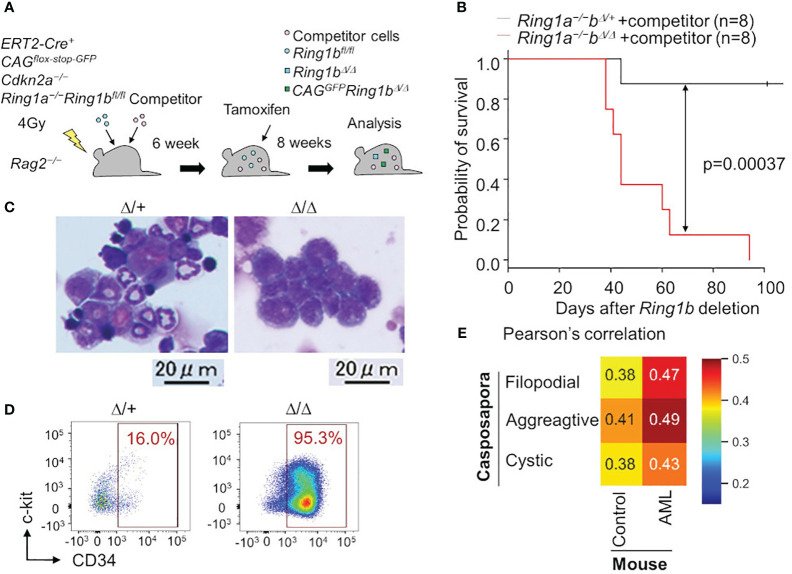
Leukemia cells are transcriptionally similar to a unicellular organism. **(A)** *Ring1a/b* were selectively deleted in blood cells using bone marrow chimera mice and the ERT2-Cre system. **(B)** Survival curve with Kaplan-Meier plots of BM transplantation into sublethally irradiated *Rag2^−/−^* mice with competitor cells. Black and red lines show survival curve of control (*Ring1a−/−Ring1b^Δ/+^*) and *Ring1a/b*-deleted (*Ring1a^−/−^Ring1b^Δ/Δ^*) mice on a *Cdkn2a^−/−^* background, respectively. Statistical significance of differences between the survival rates was calculated with the Log-rank test. **(C, D)** Wright-Giemsa stain of BM smears **(C)** and flow cytometric profiles of whole GFP^+^CD11b^+^ BM cells **(D)** obtained from control and Ring1a/b-deleted mice with competitor cells. **(E)** Pearson’s correlation values between Capsaspora, normal myeloid cells (control) and AML cells.

In the above-mentioned study, we also found that polycomb complexes maintain various blood lineages (T cell, B cell, erythrocyte, and platelet) by repressing phagocyte programs, and that disruption of polycomb complexes led to evolutionary reversion of hematopoiesis; blood in *Ring1a/b* deleted mice was occupied with monocyte/macrophage lineage cells. These findings suggest that various nonphagocytic lineages have evolved from primordial monocytes/macrophages by repressing phagocytic programs with polycomb complexes. Furthermore, these *Ring1a/b*-deleted monocyte/macrophage lineage cells looked like immature monoblasts with CD34 expression, and mice bearing these monoblasts died within a few months, indicating that these were leukemic cells (**Figures 2A-D**). This is in line with other studies in which disruption of certain polycomb complexes caused AML (54–56).

Surprisingly, we found that *Ring1a/b* KO AML cells were transcriptionally more similar to a eukaryotic unicellular organism, *C. owczarzaki*, than to normal myeloid cells (**Figure 2E**). We evaluated transcriptional similarities by calculating Pearson’s correlation values between mouse normal/leukemic cells and *C. owczarzaki*. AML cells showed higher similarities (correlation values) to all the three stages of *C. owczarzaki*. Thus, malignant tumor cells transcriptionally resemble unicellular organisms; thus, support for the hypothesis that tumorigenesis has an aspect of evolutionary reversion has become more robust. Although another possibility remains, that disruption of polycomb complexes independently contributes to tumorigenesis as well as evolutionary reversion in hematopoiesis, it is at least reasonable to think that tumorigenesis and phylogenetics have a deep relationship. The fact that histone modification by polycomb complexes is a ubiquitous mechanism also makes this hypothesis more probable (57). Other epigenetic mechanisms such as cohesion are also known to be involved in tumorigenesis (58), thus tumorigenesis, phylogenetics, and epigenetics may have a close relationship. The aspect of phylogenetics is not yet well-accepted in cancer research, therefore, integrating these three research areas is likely to bring about new developments.

**Figure 3:**
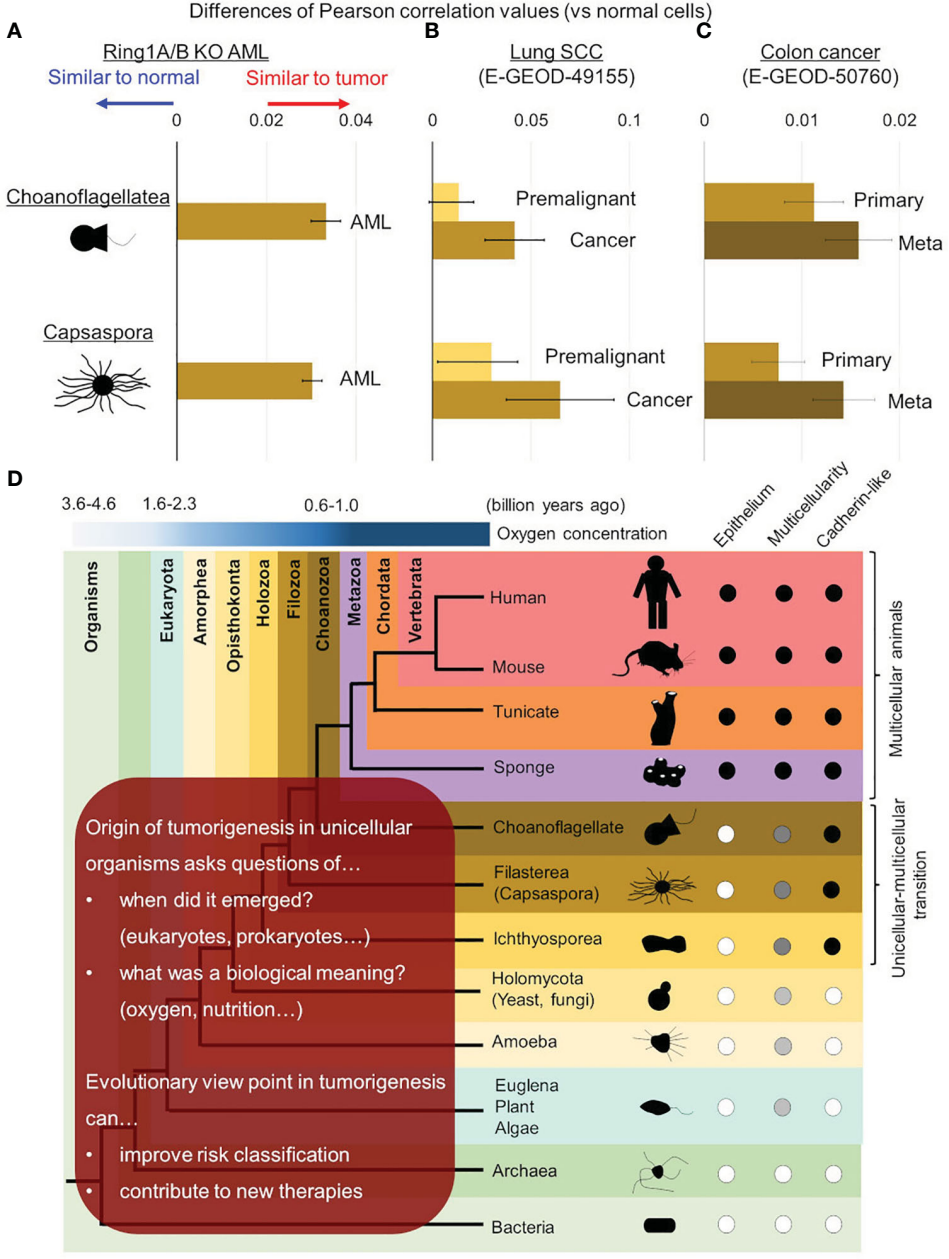
Evolutionary reversion in solid tumors and tumor progression. Differences of similarities (Pearson’s correlation values) between normal/tumor cells and unicellular organisms were calculated in mouse *Ring1a/b* deleted AML cells **(A)**, lung cancer **(B)**, and colon cancer **(C)**. **(B)** In lung cancer, similarities with unicellular organisms were compared between premalignant cells and cancer cells. **(C)** In colon cancer, similarities with unicellular organisms were compared between primary lesions and metastatic lesions. **(D)** Further investigations are required to reveal origin of tumorigenesis in detail and its biological meaning in ancestral unicellular organisms. Such findings can contribute to overcoming malignant tumors by improving risk classification and developing new therapies.

In addition to the previous study, we performed cross species analysis adding solid tumor cells and another unicellular organism, choanoflagellate (*Salpingoeca rosetta*), in order to validate our hypothesis of evolutionary reversion in tumorigenesis. In this analysis, AML cells, lung cancer cells, and colon cancer cells were examined and all of them showed higher similarities to both *C. owczarzaki* and *S. rosetta* (**Figure 3**). We also addressed the issue of traditional cancer evolution from our evolutionary standpoint. In lung cancer, similarities to unicellular organisms were also high in premalignant cells, and it increased along with tumor progression to cancer (**Figure 3B**). In colon cancer, similarities to unicellular organism got higher along with progression from primary lesion to metastatic lesion (**Figure 3C**). These data suggested a linear relationship between tumor progression and evolutionary reversion.

## Further implications of evolutionary aspects in tumorigenesis

It is now strongly suggested that tumorigenesis has an aspect of evolutionary reversion, but many issues are remained to be clarified (**Figure 3D**). The first issue is when origin of genetic program of tumorigenesis was emerged in ancestral unicellular organisms, because the evolutionary history from the initial organisms to multicellular animals was so long (3 billion years) (33, 59, 60). Although the origin of tumorigenesis may have emerged all at once in an ancestral organism, it is more probable that it emerged step by step during evolution from prokaryotes to unicellular relatives of animals. Further investigations adding various unicellular organism, such as bacteria, archaea, euglena/algae/plants, amoeba, and yeast/fungi, will help us to trace the origin of tumorigenesis more accurately. When the origin of tumorigenesis is traced back to unicellular ancestors, another question emerges; what is a biological meaning or a merit to acquire it in the ancestors? There are many hypotheses worth considering, but oxygen concentration may be essential. As for oxygen concentration, it is well known that tumor microenvironment is hypoxic (24, 61, 62), and it is also suggested that ancient unicellular organisms inhabited hypoxic environment and adjusted to the novel environment with high oxygen (63, 64) (**Figure 1**). Some ancient genetic program of tumorigenesis may have emerged in such organisms and have brought merits to survive in both of hypoxic and hyperoxic environments. Although such event was beneficial for the ancestral unicellular organisms, risk of tumorigenesis has been inherited to their progenies, and the risk turned out as malignant tumors in animals including human beings. While multicellular animals or their unicellular relatives inherited such dangerous programs, they acquired other genes to control tumorigenesis. These were origins of tumor-suppressor genes, and cell-cell adhesion should have been one of them. Ancestors of animals acquired a cadherin-like protein during evolution from unicellular organisms to multicellular animals (37). This enabled them to create stiff cell-cell adhesion and to form epithelium and multicellularity. Animals have acquired many other cadherin-like proteins including FAT family proteins throughout their evolution, and such proteins have strengthened epithelial cell identity of adhesion. This has worked as safeguard for tumorigenesis preventing premalignant cell from disengaging from epithelium. In other words, losing multicellularization-related genes has been one of the steps of tumorigenesis.

We further argued that evolutionary aspect can contribute to improving clinical outcomes of patients suffering malignant tumors (**Figure 3D**). In short term, similarities to unicellular organism can make risk-classification more accurate. For example, patients baring tumor cells highly similar to unicellular organism can show worse outcomes, and more intensive therapeutic strategy may overcome the poor prognosis. In long term, evolutionary aspects in tumorigenesis including biological meaning of origin of tumorigenesis can give us a hint to control them and to develop new therapeutic agents or methods. Evaluating effects of agents against unicellular organisms, especially unicellular relatives of animals (e.g., *C. owczarzaki* and *S. rosetta*) will give us informative implications.

In conclusion, cross-species comparisons of transcriptome data provide new insights into tumorigenesis: evolutionary reversion. Further investigations from the novel view point shall help human beings to overcome malignant tumors in the future.

## Author contributions

YN: Conceptualization, Data curation, Formal Analysis, Funding acquisition, Investigation, Methodology, Resources, Writing – original draft, Writing – review & editing. HK: Conceptualization, Funding acquisition, Project administration, Resources, Supervision, Writing – original draft, Writing – review & editing.
