# Supplementary material for: Evolutionary reversion in tumorigenesis
Source: Front Oncol. 2023 Nov 7;13:1282417. doi: 10.3389/fonc.2023.1282417 (PMC10662060; doi:10.3389/fonc.2023.1282417)
Supplement: Supplementary file 1 [file DataSheet_1.docx]

Supplementary Material

# Supplementary Data

**Methods**

**Data availability**

For comparison of gene expression profiles among different species, public data of human cancer cell (E-GEOD-49155 and E-GEOD-50760 at EMBL-EBI), mouse AML cells (DRA014437 at DNA Data Bank of Japan), choanoflagellate, and Capsaspora in previous reports were analyzed (Supplementary Table 1) ([36](#_ENREF_36), [38](#_ENREF_38), [46](#_ENREF_46)).

**Cross-species transcriptomic comparison**

For the cross-species comparison in the previous studies (48), we first identified homologs in the proteomes of *Mus musculus*, *Ciona intestinalis*, *Amphimedon queenslandica*, and *Capsaspora owczarzaki* using the OrthoFinder (Supplementary Figure 1A, B) (47). Amino acid sequences were obtained from the Ensembl and other databases and previous reports (Supplementary Table 1) (37, 38, 40, 43, 65-67). Instead of default algorithm of Diamond, we used MMseqs2 for identifying homologs. Orthogroups commonly conserved across the four species were selected and used for cross-species comparison. If one orthogroup contained two or more homologs in one species, the total amount of TPM values of the homologs was used for TPM value of the orthogroup. We then normalized TPM values only for the subset of conserved orthogroups, transformed them to log2 (TPM +1). Pearson’s correlation values were calculated based on the log2 (TPM+1) values. The PC analyses of the expression data was performed using scikit-learn.

As for original data in this article (Figure 3), we identified homologs adding proteomes of *Homo sapiens*, *Danio rerio*, *Branchiostoma lanceolatum*, *Strongylocentrotus purpuratus*, *Acanthaster planci*, *Octopus bimaculoides*, *Drosophila melanogaster*, *Nematostella vectensis*, *Trichoplax adhaerens*, *Mnemiopsis leidyi*, and *Salpingoeca rosetta*. In this analysis we compared gene expression profiles based on all the Orthogroups including species unique genes. We normalized TPM values only for protein coding genes. Then, the total amounts of TPM values of the homologs were used for TPM value of the Orthogroups, and they were transformed to log2 (TPM+1) values.

**Statistical analysis**

Survival rates were estimated using Kaplan-Meier methods and compared using Log-rank tests. Statistical analyses for survival rates were performed using EZR 1.40 (Jichi Medical University, Japan) (68). Statistical analyses for correlation of gene expression levels were performed using Anaconda 4.10.1 and Jupyter Notebook 6.3.0.

**References for Supplementary Material**

65. Hall MR, Kocot KM, Baughman KW, Fernandez-Valverde SL, Gauthier MEA, Hatleberg WL, et al. The crown-of-thorns starfish genome as a guide for biocontrol of this coral reef pest. Nature. (2017) 544:231-4. doi: 10.1038/nature22033

66. Sebe-Pedros A, Chomsky E, Pang K, Lara-Astiaso D, Gaiti F, Mukamel Z, et al. Early metazoan cell type diversity and the evolution of multicellular gene regulation. Nat Ecol Evol. (2018) 2:1176-88. doi: 10.1038/s41559-018-0575-6

67. Sebe-Pedros A, Saudemont B, Chomsky E, Plessier F, Mailhe MP, Renno J, et al. Cnidarian Cell Type Diversity and Regulation Revealed by Whole-Organism Single-Cell RNA-Seq. Cell. (2018) 173:1520-34 e20. doi: 10.1016/j.cell.2018.05.019

68. Kanda Y. Investigation of the freely available easy-to-use software 'EZR' for medical statistics. Bone Marrow Transplant. (2013) 48:452-8. doi: 10.1038/bmt.2012.244

# Supplementary Figures and Tables

## Supplementary Figures


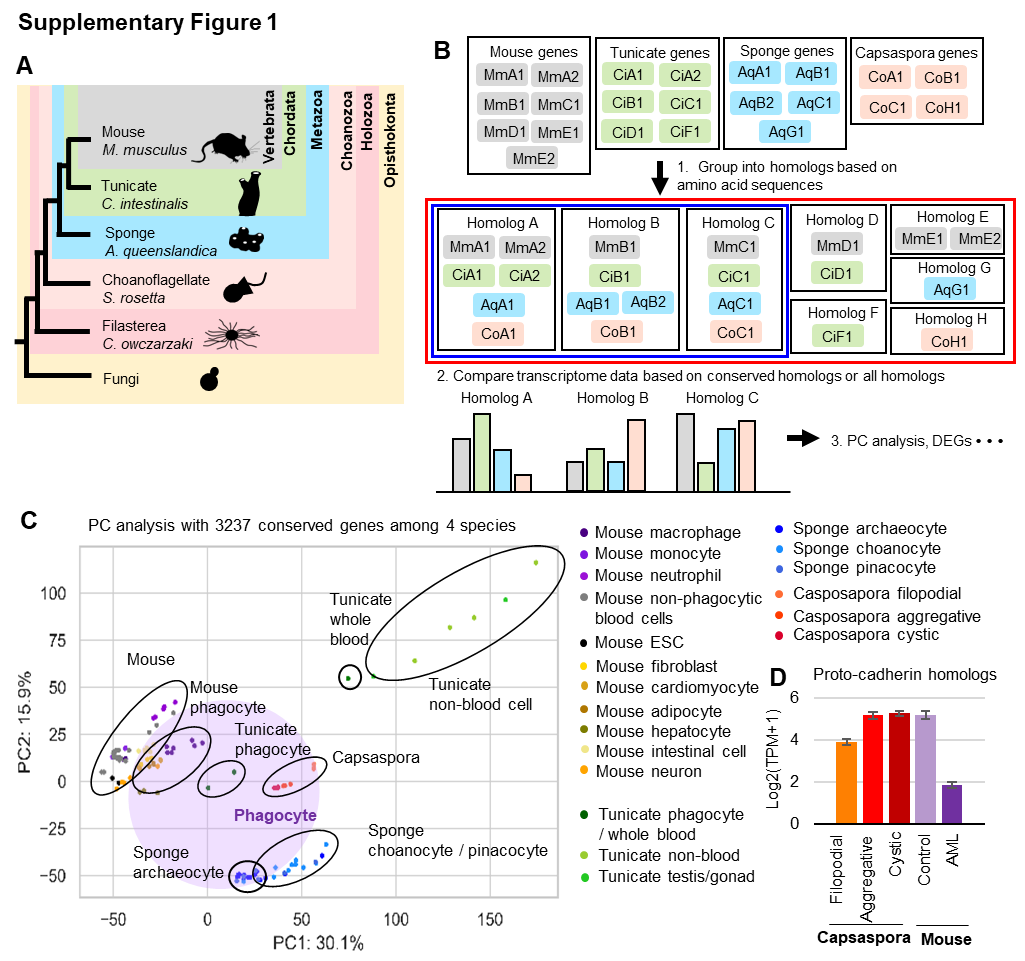


**Supplementary Figure 1.** Cross-species comparison of transcriptome data

A) Phylogenetic tree of animals and eukaryotic unicellular organisms. In our recent study (48), transcriptome data of 4 species (mouse, tunicate, sponge, and Capsaspora) were compared. (B) Method for cross-species comparison of transcriptome data. 1. Using the OrthoFinder algorithm, all protein-coding genes were grouped into homologs based on their amino acid sequences. 2. Expression levels of homologs conserved among the analyzed species (blue line, Figure 2E and Supplementary Figure 1C, D) or all homologs (red line, Figure 3A-C) were compared. 3. Transcriptomic analyses such as principal component (PC) analysis and differentially expressed genes (DEGs) can then be performed. (C) PC analysis of cross-species transcriptomic analysis of the four species. (D) Expression levels of proto-cadherin and FAT homologs in Capsaspora, normal myeloid cells (control) and Ring1a/b-deleted myeloid leukemia cells (AML).
